# Fossilized bioelectric wire – the trace fossil *Trichichnus*

**DOI:** 10.5194/bg-12-2301-2015

**Published:** 2015-04-16

**Authors:** M. Kędzierski, A. Uchman, Z. Sawlowicz, A. Briguglio

**Affiliations:** 1 Institute of Geological Sciences, Jagiellonian University, Oleandry 2a, 30-063 Kraków, Poland; 2 Institut für Paläontologie, Universität Wien, Geozentrum, Althanstrasse 14, 1090 Vienna, Austria; 3 Faculty of Science, Department of Petroleum Geoscience, Universiti Brunei Darussalam, Jalan Tungku Link, Gadong BE1410, Brunei

## Abstract

The trace fossil *Trichichnus* is proposed as an indicator of fossil bioelectric bacterial activity at the oxic–anoxic interface zone of marine sediments. This fulfils the idea that such processes, commonly found in the modern realm, should be also present in the geological past. *Trichichnus* is an exceptional trace fossil due to its very thin diameter (mostly less than 1 mm) and common pyritic filling. It is ubiquitous in some fine-grained sediments, where it has been interpreted as a burrow formed deeper than any other trace fossils, below the redox boundary. *Trichichnus*, formerly referred to as deeply burrowed invertebrates, has been found as remnant of a fossilized intrasediment bacterial mat that is pyritized. As visualized in 3-D by means of X-ray computed microtomography scanner, *Trichichnus* forms dense filamentous fabric, which reflects that it is produced by modern large, mat-forming, sulfide-oxidizing bacteria, belonging mostly to *Thioploca*-related taxa, which are able to house a complex bacterial consortium. Several stages of *Trichichnus* formation, including filamentous, bacterial mat and its pyritization, are proposed to explain an electron exchange between oxic and suboxic/anoxic layers in the sediment. Therefore, *Trichichnus* can be considered a fossilized “electric wire”.

## 1 Introduction

Bioelectric bacterial processes are omnipresent phenomena in the oxic–anoxic transition zone of recent marine sediments (see Nielsen and Risgaard-Petersen, 2015, for review). It is intriguing that they have not so far been recognized from the geological past as it was suggested by Risgaard-Petersen et al. (2012). One reason could be that effects of such bacterial processes, leading to oxygenation of the anoxic sediments on the sea floor, are eventually destroyed by subsequent bioturbation (Malkin et al., 2014). We propose here that the usually pyritized marine trace fossil *Trichichnus* Frey (1970) can be interpreted as a fossil record of complex bioelectrical operations resulting from bacterial activities in the oxygen-depleted part of marine sediments. *Trichichnus* is a branched or unbranched, straight to winding, hair-like cylindrical structure, mostly 0.1–0.7 mm in diameter, commonly pyritized, oriented at various angles (mostly vertical) with respect to the bedding. The common pyritization is a particular feature of *Trichichnus*, which is generally absent in other trace fossils. *Trichichnus* is reported from both shallow- and deep-sea, mostly fine-grained sediments (Frey, 1970; Wetzel, 1983). It ranges from the Cambrian (Stachacz, 2012) to the Holocene (Wetzel, 1983). There are distinguished *Trichichnus appendicus* displaying thin appendages. *T. linearis* or *T. simplex* probably differs only in the presence of a lining, which likely is diagenetic in origin (see Uchman, 1999, for review). *Trichichnus* is common in sediments in which pore waters were poorly oxygenated (McBride and Picard, 1991; Uchman, 1995) and is considered to be one of the first trace fossils recording colonization of the sea floor after improvement of oxygenation, penetrating sediments below the redox boundary and having a connection to oxygenated waters on the sea floor (Uchman, 1995). It belongs to the ethological category chemichnia distinguished for trace fossils produced by organisms feeding by means of chemosymbiosis (Uchman, 1999).

*Trichichnus* was previously interpreted as a deep-tier burrow produced by unknown opportunistic invertebrates in poorly oxygenated sediments (McBride and Picard, 1991; Uchman, 1995). It has also been compared to an open burrow in modern deep-sea sediments more than 2 m long and no more than 0.5 mm thick (Thomson and Wilson, 1980; Weaver and Schultheiss, 1983). The sipunculid worm *Golfingia* has been considered as a possible modern example of trace makers of very narrow and long tubes similar to *Trichichnus* (Romero-Wetzel, 1987). However, 3-D microCT scanner images of *Trichichnus* presented in this paper reflect fabric produced by modern, large, sulfur bacteria. Therefore, a new interpretation of *Trichichnus* is possible by its comparison to modern sulfur bacteria such as *Thioploca* spp. and their postmortem history. Moreover, we propose a biogeochemical model of *Trichichnus* assisted electron transfer between different redox zones of the sediment.

## 2 Material and methods summary

The morphology and chemistry of *Trichichnus* from several samples, represented by Albian turbiditic marly mudstones (Silesian Nappe, Polish Carpathians); Valanginian–Hauterivian pelagic marlstones (Kościeliska Marl Formation, the Tatra Mountains) and Eocene silty shales (Magura Nappe, Polish Carpathians), were studied using a scanning electron microscope with field emission (FE-SEM, Hitachi S4700), equipped with EDS (Noran Vantage), which allows the determination of chemical compounds of the examined objects. The studies on morphology and infilling of *Trichichnus* were also supported using the polarizing light microscope Nikon Eclipse Pol E-600. All of the above were carried out at the Institute of Geological Sciences of the Jagiellonian University in Kraków. Moreover, the X-ray computed microtomography (microCT) scanner (SkyScan 1173) available at the Institute of Palaeontology, University of Vienna, was used to carry out the main research on the spatial organization of the *Trichichnus* (Table 1).

All samples of trace fossil *Trichichnus* presented in the paper are housed at the Institute of Geological Sciences, Jagiellonian University, Kraków, Poland, collection of Alfred Uchman and Zbigniew Sawlowicz. No permits were required for the described study, which complied with all relevant regulations.

## 3 Results

**Figure 1:**
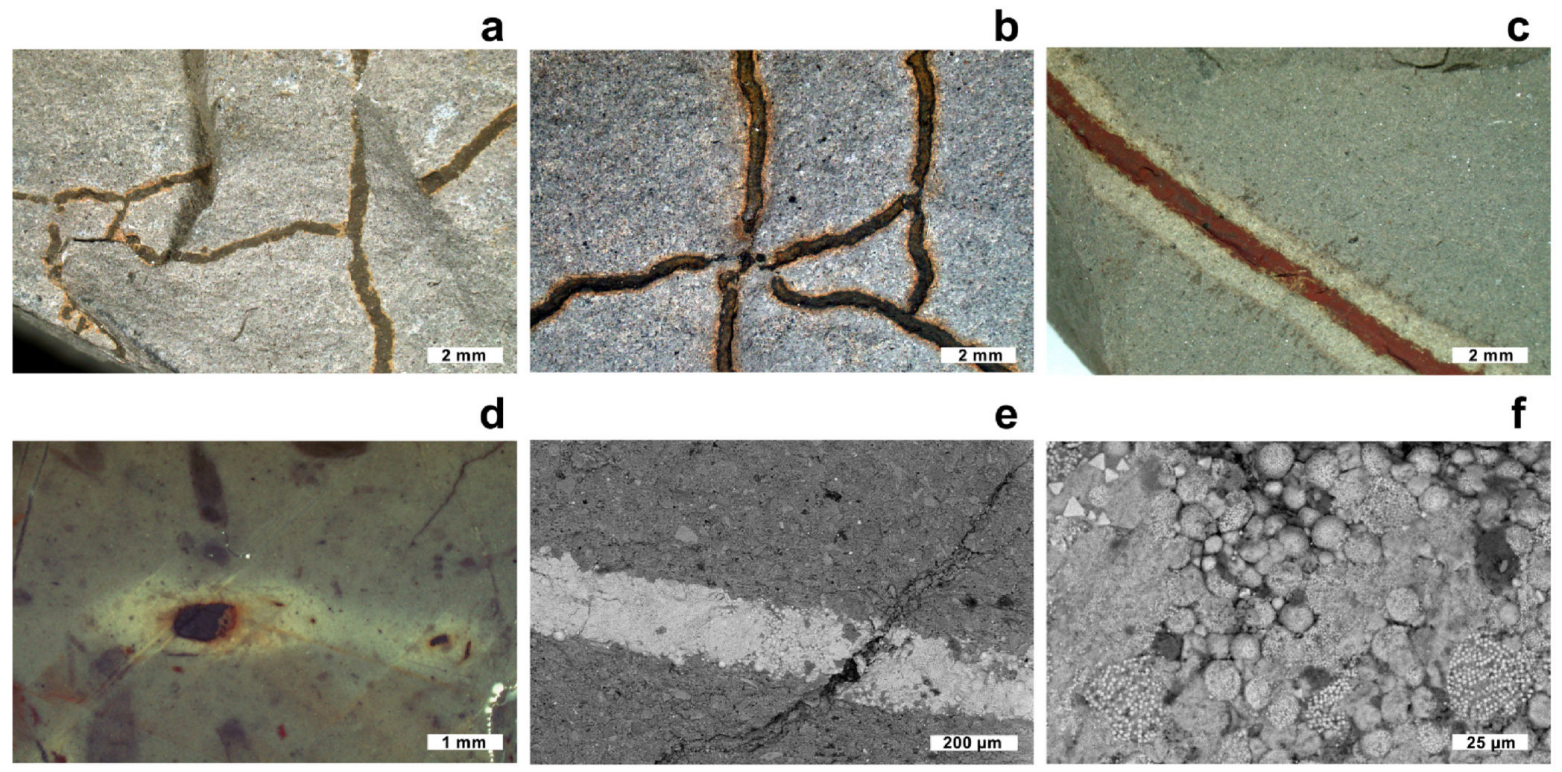
Macro- and microimages of *Trichichnus* in the rock fractures and slabs: **(a–c)** Albian turbiditic marly mudstones, Kozy, Polish Carpathians; **(d)** Valanginian–Hauterivian pelagic marlstones. Dolina Kościeliska valley, Tatra Mountains, Poland **(e)**, **(f)** Eocene silty shales, Zbludza, Polish Carpathians. **(a)**–**(b)** Subhorizontal network of *Trichichnus*. **(c)**–**(d)** Halo (diffusion zone of iron (oxi)hydroxides) around horizontal **(c)** and vertical **(d)** *Trichichnus*. **(e)** Infilling of a fragment of *Trichichnus* with abundant pyrite framboids and iron (oxi)hydroxides (SEM BSE). **(f)** Magnification of image **(e)**. Specimen numbers: **(a)** INGUJ147P70, **(b)** INGUJ147P71, **(c)** INGUJ147P72, **(d)** INGUJ155P20, **(e)**–**(f)** INGUJ144P159.

With the naked eye or under light microscope *Trichichnus* is easily distinguished in all rocks samples mainly by its different color (Fig. 1a–d). Macroscopically *Trichichnus* is similar in all samples and shows a light halo around the trace, except in burrows without iron mineral infillings (Fig. 1c and d). Neither the length nor width of the trace depends on the type of the host rock. The boundary between the trace fossil and the rock is usually sharp (Fig. 1e).

The SEM studies revealed that majority of the studied *Trichichnus* is filled with framboids and framboidal aggregates (Fig. 1e and f). Framboidal forms are composed of iron sulfides (most probably pyrite), iron oxi/hydroxides or a mixture of both. The microcrystals building framboids are generally well-developed and their sizes are usually about 1–2 μm, generally dependent on the size of the framboid. Most of the microcrystals in framboids are closely packed. SEM-EDS study shows that the colorful halo around traces does not reveal any chemical differences from the composition of the surrounding rock. It is likely that the amount of iron minerals in the halo is below the detection limit of the EDS method. Apart from the iron minerals, subordinate amounts of several other minerals, generally corresponding to the host rock, such as calcite, quartz and gypsum, are observed.

**Figure 2:**
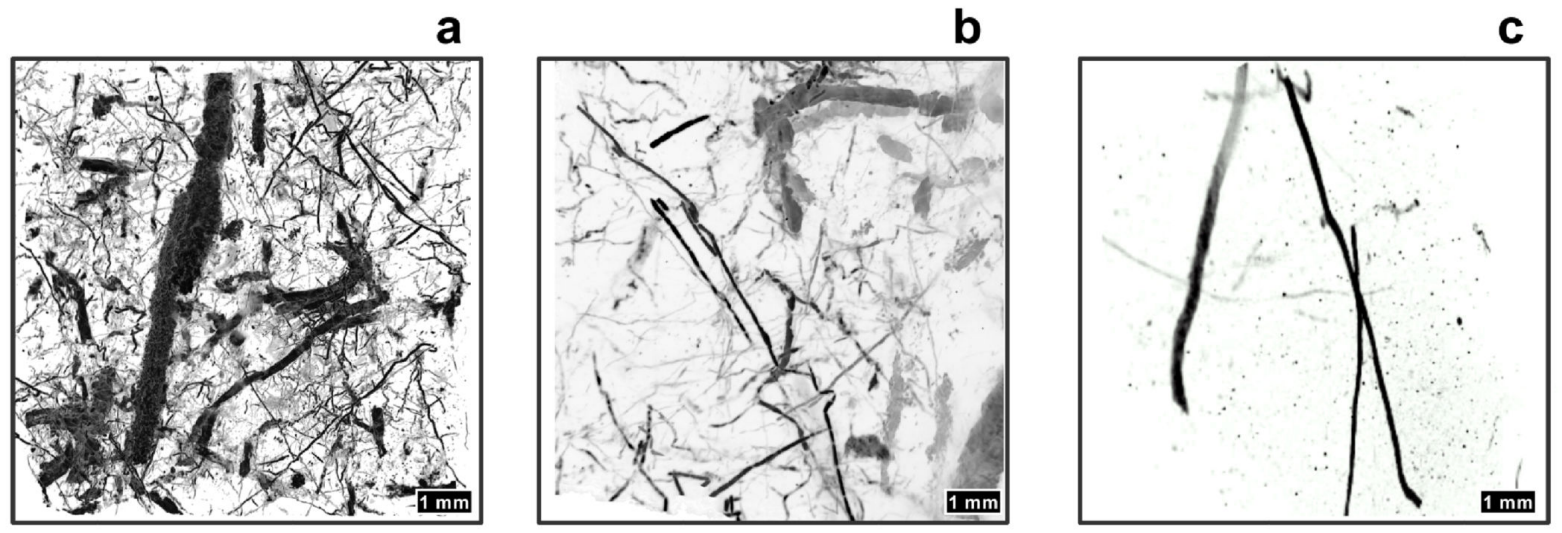
X-ray computed microtomography scanner images of different parts of *Trichichnus* spatial complex, Oligocene, Skole Nappe, Polish Flysch Carpathians: **(a)** Dense *Trichichnus* fabric, similar to the upper part of the *Thioploca* mat system. **(b)** *Trichichnus* fabric comparable with the middle part of the *Thioploca* mat system. **(c)** *Trichichnus* fabric depicting the lower part of the *Thioploca* mat system.

MicroCT scans allowed three-dimensional visualization of several *Trichichnus* embedded in a dark mudstone from the Oligocene Menilite Formation in the Western Carpathians, Poland. The density difference between the sediment matrix and the pyritic trace fossil fillings allowed extremely accurate three-dimensional visualization (Fig. 2). The results show a very dense fabric composed of *Trichichnus* fillings of variable size and orientation, which are only partly visible on a sample surface. Since the resolution of the 3-D model here presented is 9.97 μm, all the structures larger than 10 μm are visible and renderable. The distribution of diameters of the traces displays two major peaks: 600 μm for the thickest traces and 90 μm for the very thin ones (Fig. 2a; see also the Supplement).

The *Trichichnus* morphology seen in 3-D microCT scanner image (Fig. 2a–c) is similar to structures produced by filamentous bacteria (e.g., Pfeffer et al., 2012) or to large, mat-forming, filamentous sulfur bacteria, e.g., *Thioploca* spp. (Fossing et al., 1995, their Fig. 3a; Schulz et al., 1996, their Fig. 8; Jørgensen and Gallardo, 1999, their Fig. 5; see also Heutell et al., 1996). The microCT scanner images also reveal different spatial organizations, density, diameters and shapes of the *Trichichnus*. These correspond to different parts of vertical system of the *Thioploca* mat in sediment. Also the density of *Trichichnus* (Fig. 2a), which constitutes 3.1 % of the whole scanned sediment volume, is comparable to the *Thioploca* mat in their upper shallow subsurface part. Other 3-D scanner images (Fig. 2b and c) show lowered density of *Trichichnus,* comparable to middle and bottom parts of the *Thioploca* filamentous mat spatial system.

## 4 Discussion

Three-dimensional reconstructions of *Thioploca* spp. (Fossing et al., 1995; Schulz et al., 1996; Jørgensen and Gallardo, 1999) resemble *Trichichnus* in the 3-D scanner images. Moreover, the common pyritization of *Trichichnus* is related to sulfate-reducing bacteria, like *δ*-proteobacteria *Desulfovibrio* spp., which can co-occur with sulfur-oxidizing bacteria, such as *Beggiatoa* or *Thioploca* (e.g., Jiang et al., 2012). Therefore, we consider *Thioploca*-like, mat-forming, large sulfur bacteria and related filamentous sulfide-oxidizing bacteria (see Teske et al., 1995) as a trace maker of *Trichichnus* tunnels and their spatial organization. The *Thioploca*-like mat, usually hosting other bacteria or small protists (e.g., Buck et al., 2014), combined with the postmortem history of sulfur bacterial mat systems, refer to the idea of bioelectrochemical systems (BESs), including microbial fuel cells (MFCs) and biogeobatteries (e.g., Naudet et al., 2003, 2004; Naudet and Revil, 2005; Linde and Revil, 2007; Ntarlagiannis et al., 2007; Logan, 2009; Teske et al., 2009; Nielsen and Girguis, 2010; Revil et al., 2010; Borole et al., 2011; Hubbard et al., 2011; Risgaard-Petersen et al., 2014; Nielsen and Risgaard-Petersen, 2015, for review).

In biogeobattery systems, bacteria are interconnected cells to cells or cells to mineral surfaces via electrically conductive appendages – bacterial pili (nanowires) – making a complex electroactive network (biofilm) (Ntarlagiannis et al., 2007; Revil et al., 2010; Borole et al., 2011; Reguera et al., 2005; Gorby et al., 2006; El-Naggar et al., 2010; Nielsen et al., 2010; Risgaard-Petersen et al., 2014; Nielsen and Risgaard-Petersen, 2015). The electron transport in BESs may be realized in various ways, including transfer through long bacterial nanowires or along biofilm matrix (Lovley, 2008). Its magnitude and duration mostly depend on atmospheric oxygen (electron acceptor) availability that generates electrical self-potentials in the sediments between oxidized and anoxic zones (see Ntarlagiannis, 2007; Risgaard-Petersen et al., 2014). Studies reporting extracellular bioelectric current concerned dissimilatory metal-reducing bacteria, such as *Geobacter sulfurreducens, Shewanella oneidensis* MR-1 and the thermophilic, fermentative bacterium *Pelotomaculum thermopropionicum* or the oxygenic phototrophic cyanobacterium *Synechocystis* (Borole et al., 2011). Natural conductive minerals (e.g., magnetite, greigite – an intermediate stage for pyrite formation) can facilitate electron transfer between different species of microbes. Presumably microbes should preferentially use mineral particles, discharging electrons to and accepting them from mineral surfaces, in particular for long-distance electron transfer (Kato et al., 2012). The interaction between minerals and bacteria can be quite complex as Kato et al. (2013) showed that, for example, *G. sulfurreducens* constructed two distinct types of extracellular electron transfer (EET) paths, in the presence and absence of iron-oxide minerals. It is worth noting that large-scale sulfide ores, where pyrite is commonly a main component, have been regarded as geobatteries for many years and used by geophysicists for exploration. In natural electrochemical processes an ore serves as a conductor to transfer the electrons from anoxic to oxic environments (Sato and Mooney, 1960; Naudet et al., 2003). Newer reports show that coupling of geochemical reactions between shallower and deeper layers of the sediment can be also realized by vertical centimeter-long filaments of multicellular bacteria of the family Desulfobulbaceae, the so-called cable bacteria (Pfeffer et al., 2012; Risgaard-Petersen et al., 2014; Schauer et al., 2014). Nevertheless, only giant sulfur bacteria, so-called “macro-bacteria”, are able to produce mat spatial system in the scale of *Trichichnus*. Macro-bacteria of Beggiatoacea family are considered to be the most direct competitors to cable bacteria at the interface of oxic–anoxic zone of marine sediments (see Nielsen and Risgaard-Petersen, 2015). We propose *Thioploca* spp., one of the genus of Beggiatoacea family (see Salman et al., 2011), as a trace maker of *Trichichnus*.

Large communities of the sulfur bacteria *Thioploca* spp. produce the filaments consisting of thousands of cells that can penetrate 5–15 cm down into the sediment. These filaments (trichomes), bundled in common slime sheaths, make a dense bacterial mat, where the density decreases with depth leaving single sheaths in the deepest part of the spatial system of the mat. The *Thioploca* sheaths, rarely branched, extend in different directions, mostly vertically. One sheath can accommodate up to 100 filaments of 15–40 μm in diameter each, giving about 4 mm for the maximum diameter of the sheath (Fossing et al., 1995; Schulz et al., 1996). *Thioploca* trichomes gliding within sheaths can migrate through several redox horizons, coupling the reduction of nitrate in the overlying waters and the oxidation of dissolved sulfide in the sulfate reduction zone (Fossing et al., 1995; Heutell et al., 1996). Therefore, the sheaths are used as communication tubes and enable the sulfur bacteria to switch vertically between nitrate and sulfide (electron acceptor and donor, respectively). This sulfide oxidization mechanism leads to accumulation of elemental sulfur globules in the bacterial cells. Moreover, *Thioploca* trichomes may leave their sheaths and move into other sheaths; thus, one sheath can be occupied by different species (Jørgensen and Gallardo, 1999). Empty sheaths were found even at > 20 cm depth into the sediment. In contrast, the sheaths were not found shallower than a depth of a few centimeters in the sediments. Therefore, the surface and topmost part of the *Thioploca* mat system is formed by single bacterial filaments only (Schulz et al., 1996). However, brackish/freshwater *T. ingrica* may not form any mats at the sediment surface, showing biomass peaks 4–7 cm below the sediment surface (Høgslund et al., 2010).

The *Thioploca* sheaths – either filled with trichomes or abandoned – may be closely associated with other filamentous sulfate-reducing bacteria like *Desulfonema* spp. (Jørgensen and Gallardo, 1999; Teske et al., 2009). *Thioploca* spp. are known from both marine and lake environments. Nevertheless, their mass occurrences are related to high organic production and oxygen depletion, e.g., the Chile and Peru offshore upwelling system (Jørgensen and Gallardo, 1999).

Pyritized organic remains are common in the sedimentary record, and various mechanisms of fossil pyritization have been described (e.g., Canfield and Raiswell, 1991; Briggs et al., 1996). Apart from the similarity between the *Trichichnus* fabric and spatial organization of the *Thioploca* mat system, the common pyritization of *Trichichnus* also indicates its close relation to the sulfate-reducing bacteria. Schulz et al. (2000) observed that empty sheaths of *Thioploca* from the Bay of Conception during autumn/wintertime were stained with iron sulfides. As the most abundant metal sulfide in nature, pyrite FeS_2_ has a major influence on the biogeochemical cycles of sulfur and iron, and through its oxidation also of oxygen. There have been several important reviews on sedimentary pyrite formation (e.g., Berner, 1970; Schoonen, 2004; Rickard and Luther, 2007, and references therein), specifically on iron sulfide framboids (e.g., Love and Amstutz, 1966; Raiswell, 1982; Wilkin and Barnes, 1997; Sawłowicz, 2000; Ohfuji and Rickard, 2005, and references therein). It is important to stress that at low temperature pyrite growth is usually preceded by the formation of unstable iron monosulfides, such as mackinawite and greigite. The latter is the ferrimagnetic inverse thiospinel of iron. Pyrite, like other sulfide minerals, is a semiconductor determined to be “semi-metallic”, but its conductivity in different admixtures varies widely between 0.02 and 562 (Ωcm)^−1^ (Rimstidt and Vaughan, 2003, and references therein). Pyrite has a great potential in both interaction with microorganisms and electron transfer as its range of morphological, chemical and electric characteristics is quite large. Both iron and sulfur, necessary for pyrite formation, are pivotal for microbes. For instance, more than 50 % of reduced sulfate in sediments inhabited by *Thioploca* off the Chilean coast is accumulated in the pyrite pool (Ferdelman et al., 1997). Iron is an important carrier of electrons in microbial ecosystems (Wielenga et al., 2001), and bacteria play important role in different processes of iron oxidation and reduction (Weber et al., 2006). Interestingly, some Firmicutes bacteria species such as *Desulfitobacterium frappieri* are capable of both reducing Fe^3+^ with H_2_ as an electron donor and oxidizing Fe^2+^ with nitrate as an electron acceptor (Shelobolina, 2003). In sedimentary environments the major source of sulfur incorporated into iron sulfides is H_2_S or HS^−^ resulting from bacterial sulfate reduction. Sulfate-reducing bacteria (SRB) use sulfate as the terminal electron acceptor of their electron transport chain (e.g., Widdel and Hansen, 1992). SRB are usually regarded as strictly anaerobic, but recent investigations showed that SRB are both abundant and active in the oxic zones of mats (see Baumgartner et al., 2006, for review). Iron monosulfides and pyrite, including framboids, are quite common in some microbial mats (e.g., Popa et al., 2004; Huerta-Diaz et al., 2012), but their formation is complicated and difficult to study. Nevertheless, sulfur content and sulfur speciation may not correlate with microbial metabolic processes (Engel et al., 2007). Oxidation of iron sulfides is one of the most common processes in marine sediments. For example, Passier et al. (1999) reported that in the Mediterranean sapropels the percentage of initially formed iron sulfides that were re-oxidized varied from 34 to 80 %. Oxidation of iron sulfide framboids to iron (hydro)oxides is often observed in fossils (e.g., Luther et al., 1982; Sawłowicz, 2000). There is a significant difference between oxidation of pyrite and its precursors. For example, FeS, but not pyrite, can be oxidized microbially with ${\mathrm{NO}}_{3}^{-}$ as an electron acceptor (Schippers and Jørgensen, 2002).

## 5 Conclusions and model of *Trichichnus*

**Figure 3:**
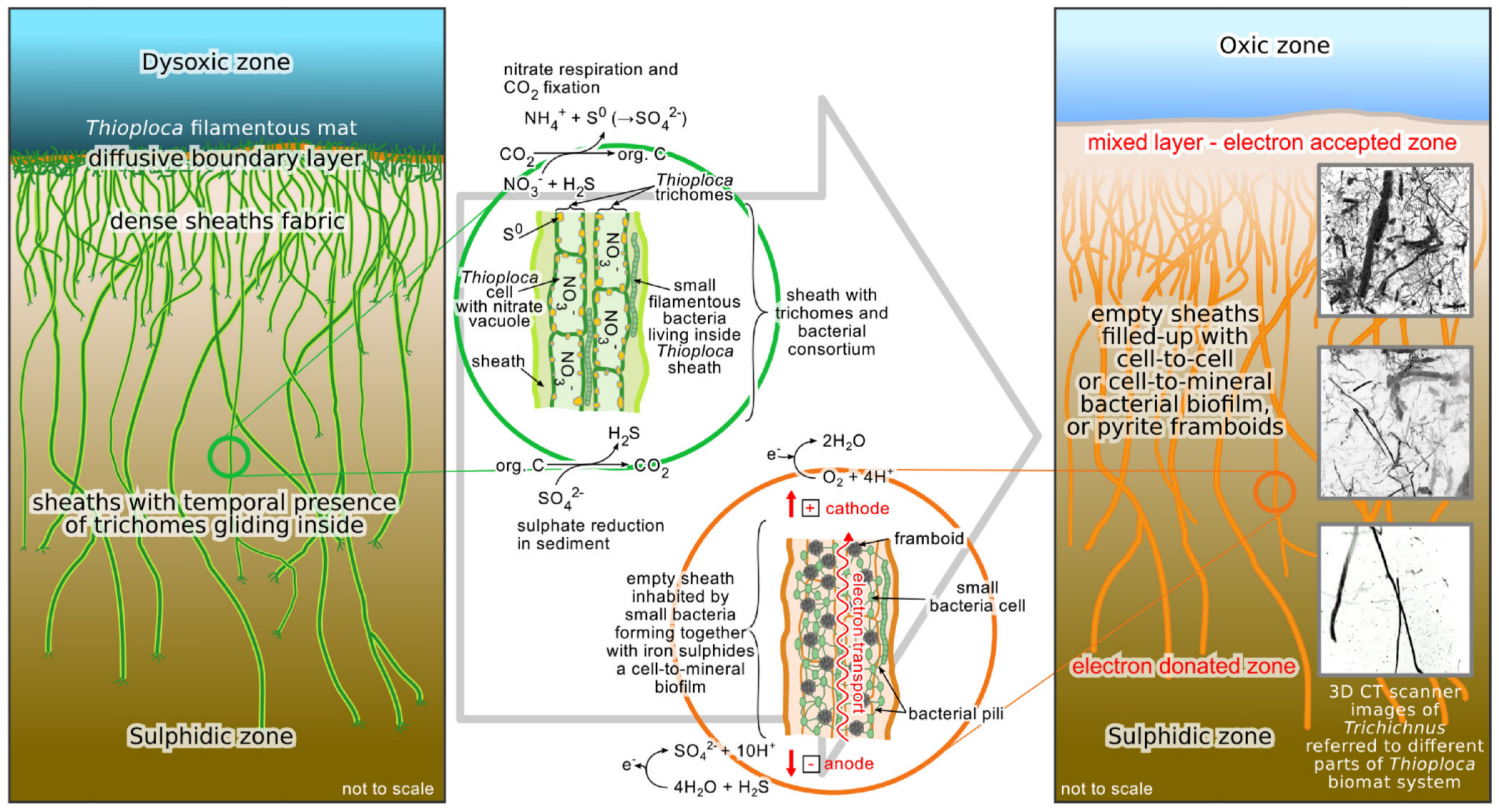
Model of the origin of *Trichichnus*. The ecology of *Thioploca* mat system and their sulfide oxidation chemistry (left). The *Thioploca* sheath system below the diffusive boundary layer forms a construction which may be inhabited by small bacteria making the conductive nanowire–pyrite framboid biofilm. This triggers the electric self-potential between the sulfidic zone and mixed layer, thus, the electron flow (right). The different part of the *Trichichnus* and the different part of the former *Thioploca* mat system are shown for comparison (right). *Thioploca* spp. nitrogen, carbon and sulfur metabolism reactions are taken from Teske and Nelson (2006); half-reactions on *Trichichnus* are adapted from Nielsen and Risgaard-Petersen (2015).

*Trichichnus* is a good example of microbiological and mineralogical collaboration, propounded earlier by Naudet et al. (2003, 2004). Its role in electron transfer is still enigmatic, but several stages can be proposed (Fig. 3). It should also be noted that the relatively large length of *Trichichnus* and its continuation through different redox zones may result in its vertical internal zonation. As suggested earlier, *Trichichnus* may reflect former slime sheaths, where large sulfur bacterial trichomes, like *Thioploca* community, move up and down. There is growing evidence (e.g., Teske et al., 2009; Prokopenko et al., 2013; Buck et al., 2014, and references therein) that *Thioploca* sheaths house a complex bacterial consortium. In our opinion it is only a matter of time before additional microbes will be found associated with *Thioploca* or their postmortem sheaths.

1. The earliest stage of the *Trichichnus* formation is related to a sulfuric microbial mat in the dysoxic zone, connecting the anoxic–sulfidic substrate. Long, filamentous bacteria structured like electric cables facilitate electron transport over centimeter distances in marine sediment (Pfeffer et al., 2012; Risgaard-Petersen et al., 2014). The filamentous multicellular, aerobic bacteria of the family Desulfobulbaceae (cable bacteria), living together with *Thioploca* in the sheath, conduct electrons through internal insulated wires from cells in the sulfide oxidation zone to cells in the oxygen reduction zone (Pfeffer et al., 2012). For *Thioploca* itself such behavior has not been proven yet (see Nielsen and Risgaard-Petersen, 2015, for review). The electric circuit is balanced by charge transport by ions in the surrounding environment (Risgaard-Petersen et al., 2014; Nielsen and Risgaard-Petersen, 2015).
2. The next stage is related to the iron sulfidization process. Microorganisms can influence both the precipitation of sulfides and their morphology. Bacterial components (e.g., cell walls) facilitate mineralization by sequestration of metallic ions from solution and provide local sites for nucleation and growth (Beveridge and Fyfe, 1985). Iron sulfides (framboids) can form within a matrix of bacteria and biopolymers both during the life-time of bacterial consortium (see MacLean et al., 2008) and after their death. It should be noted that the process of sheath infilling varies within the system. For example, larger sheaths may be abandoned by trichomes of *Thioploca* faster, compared to smaller ones (Schulz et al., 1996). The biofilm in a proto-*Trichichnus* capsule seems to be an ideal place for the formation of iron sulfides. Formation of pyrite, probably formerly ferromagnetic greigite, depends on availability of soluble Fe(II) from a surrounding sediment and sulfides (H_2_S or polysulfides). Crystals in framboids are generally closely packed, and framboids themselves often too, but when it is not the case, the bacterial pili are believed to connect dispersed conductive minerals (Revil et al., 2010). Cell-to-mineral wires support the hypothesis that the metallic conductor-like pyrite occurring in sediment might be responsible for the electron flow (see also Nielsen et al., 2010). Growth of a biofilm – developed in the mucus of the large sulfur bacteria sheath – on pyrite can enhance conductivity supporting the biogeobattery idea. The oldest microbial communities colonizing sedimentary pyrite grains were found in a ~3.4-billion-year-old sandstone from Australia (Wacey et al., 2011).
3. The process of electron transfer can continue also after termination of microbial consortia. Decomposition of organic matter inside the channel creates the local reducing microenvironment, which promotes pyrite formation (e.g., Berner, 1980; Raiswell, 1982), even when the surrounding sediment is not fully anoxic. It is tempting to propose that perhaps some iron sulfide forms resulted from a replacement of sulfur globules (stored intra- and extracellularly by chemolithoautotrophic bacteria, e.g., *Thioploca* or by purple and green sulfur bacteria; Oschmann, 2000), either inside sulfur bacteria cells or after lysis. Spheroidal crystalline aggregates representing early pyrite and subsequently replaced by iron hydroxides were found in Silurian cyanobacterial filaments (Tomescu et al., 2006). Formation of pyrite framboids via replacement of sulfur grains was also shown experimentally by Křibek (1975). A halo observed around *Trichichnus* (Fig. 1c and d) results from oxidation of pyrite or former iron monosulfide infilling the trace fossil. The oxidation stage can take place both during the earliest and later stages of diagenesis (the latter is out of the scope of this paper). Re-oxidation processes of pyrite framboids are common in tropical upwelling area, caused by bioturbation and possible contributions from sulfide-oxidizing bacteria (Diaz et al., 2012, and references therein). It would additionally expand, but also complicate, the process of electron transfer in sediments.

Summarizing, we believe that *Trichichnus* is a good example of a potential ancient place where several bioprocesses related to electron transfer in sediment could proceed. A large amount of evidence of such processes, both in the field and laboratory investigations, has been growing significantly for last few years. Development of new technologies, e.g., multipurpose electrodes which combine reactive measurements with electrical geophysical measurements (Zhang et al., 2010), opens new frontiers in monitoring microbial processes in sediments. We believe that our idea raises special attention to *Thioploca* endobionts with respect to their electron exchange along the cell-to-mineral wires. Our interpretation shows how this phenomenon could have been widespread in sedimentary environments and through the geological time.

## Supplementary Material

movie

## Figures and Tables

**Table 1:** Scanner setting data used for X-ray computed microtomography (microCT).

Scanner	SkyScan 1173
Camera pixel size	50.0 μm
Source voltage	130 kV
Source current	61 μA
Camera binning	1 × 1
Filter	Al 1 mm
Exposure	650 ms
Rotation step	0.15°
Frame averaging	30
360° rotation	on
Scan duration	14 h 22 m
Pixel size	9.97646 μm
